# Serine Protease HtrA2 from Halophilic Archeon *Haloarcula* sp. TG1: Heterologous Expression, Characterization and Immobilization

**DOI:** 10.3390/biom16030424

**Published:** 2026-03-13

**Authors:** Aslıhan Kurt-Kızıldoğan, Ömer Konuksever, Özlem Yavuz, Çiğdem Otur, Büşra Abanoz-Seçgin, Sezer Okay

**Affiliations:** 1 Department of Agricultural Biotechnology, Faculty of Agriculture, Ondokuz Mayıs University, Samsun 55139, Türkiyeozlemyavuz2228@gmail.com (Ö.Y.); cigdem.otur@omu.edu.tr (Ç.O.); abanozbusra@gmail.com (B.A.-S.); 2 Department of Vaccine Technology, Vaccine Institute, Hacettepe University, Ankara 06230, Türkiye

**Keywords:** enzyme immobilization, haloarchaea, halophilic protease, PHB

## Abstract

Halophilic proteases are valuable in industrial applications due to their resistance to harsh conditions. HtrA2 serine protease is widely distributed and conserved among eukaryotes and prokaryotes. However, HtrA2 proteases from archaea have been poorly characterized. In this study, *htrA2* from haloarcheon *Haloarcula* sp. TG1 was cloned and corresponding nucleotide and amino acid sequences were analyzed. Recombinant HtrA2 was produced in *Escherichia coli*, and biochemical properties of purified HtrA2 were characterized. HtrA2 was immobilized for the first time using polyhydroxybutyrate (PHB) nanoparticles. Additionally, potential of HtrA2 as a detergent additive was evaluated by its bloodstain removal activity. Recombinant HtrA2 showed its optimum activity at 50 °C, pH 7.0, and 3.0 M NaCl. HtrA2 activity was highly retained over wide temperature (40 to 60 °C) and pH ranges (pH 5.0 to 11.0). Moreover, various organic solvents, inhibitors and metal ions were well tolerated by the enzyme. Acetone and Fe^2+^ significantly increased HtrA2 activity, while it was not inhibited by phenylmethylsulfonyl fluoride and sodium dodecyl sulfate. Also, immobilization of HtrA2 onto PHB nanoparticles improved its reusability. Furthermore, HtrA2 successfully removed the bloodstain from cotton fabric. This comprehensive characterization of HtrA2 demonstrates that recombinant HtrA2 obtained from *Haloarcula* sp. TG1 is promising for industrial applications.

## 1. Introduction

Halophilic archaea are microorganisms living in habitats with salt concentrations between 15% and 30%, such as seas, lakes, saline soils, and some foods [1,2]. Halophilic archaea, thanks to specialized survival strategies they have developed, produce a variety of biotechnologically important biomolecules that remain stable under extreme conditions. These biomolecules include enzymes, biosurfactants, and pigments. Enzymes, in particular, are one of the most important products of microbial biotechnology [3].

Halophilic enzymes are essential components that enable halophilic archaea to survive in extremely salty environments. Their ability to remain active even when exposed to high salt concentrations, their highly specific product selectivity, their non-toxicity, and their ability to remain stable under challenging reaction conditions make them useful in various applications [4], especially in many challenging industrial processes where high salt concentrations can inhibit enzymatic production [5]. Recent studies on the purification and characterization of various halophilic enzymes, such as glycosidases, proteases, and lipases, have presented potential applications [6], particularly in areas such as biofuel production [7], food processing [8], and the biological degradation of organic pollutants [9,10].

Proteases are enzymes that break down proteins into smaller protein fragments and hydrolyze peptide bonds [11]. Protease enzymes produced by halophilic microorganisms are active under extreme conditions due to the presence of unusual features such as salt inclusion strategy, osmolyte deposition, and protein glycosylation [12,13]. Most halophilic proteases belong to the serine protease group, which has active sites containing serine amino acids, and are distinguished by their ability to remain active at high salt concentrations and form a catalytic triad between serine, aspartic acid, and histidine amino acids. Numerous studies have been conducted on serine proteases; however, the molecular mechanisms of halophilic serine proteases are still being investigated. Studies to date have shown that the optimum activity of halophilic proteases is obtained in a wide range of pH, temperature and NaCl concentrations [13,14,15,16].

HtrA2, also known as the serine protease Omi, belongs to the high-temperature requirement factor A (HtrA) family. HtrA proteases constitute a highly conserved family of serine proteases widely distributed in prokaryotic and eukaryotic organisms. Members of this family play a central role in cellular protein quality control by degrading misfolded or damaged proteins, particularly under stress conditions. In addition to their proteolytic activity, certain HtrA proteins also exhibit chaperone-like functions and participate in the regulation of cellular signaling pathways [17]. HtrA2 was initially identified as the mammalian homolog of the heat shock-induced serine proteases HtrA/DegP and DegS from *Escherichia coli*, and it is among the proteins associated with programmed cell death in eukaryotes. HtrA-like membrane-associated proteases are also common among bacteria other than *Mycoplasma*. In contrast, information about archaeal HtrA proteins is quite limited [18,19]. To our best knowledge, characterization of a recombinant HtrA2 from a haloarcheon for biotechnological applications has not been reported.

In our previous study, we characterized the extracellular proteasome of *Haloarcula* sp. TG1, an extreme haloarcheon isolated from Lake Tuz in Turkey [20]. In this study, for the first time, the HtrA2 serine protease enzyme of *Haloarcula* sp. TG1 was expressed recombinantly in *Escherichia coli* BL21 cells and purified. The enzyme was biochemically characterized by evaluating its activity and stability in the presence of different temperatures, pH levels, NaCl concentrations, organic solvents, and metal ions. Moreover, our study is the first to immobilize a haloarchaeal protease with polyhydroxybutyrate (PHB) polymer. PHB was selected as the immobilization support material because *Haloarcula* sp. TG1, the source of HtrA2 in this study, is also a natural PHB producer [21]. Therefore, we wanted to use a naturally occurring biopolymer of *Haloarcula* sp. TG1 for the immobilization of the protease originating from the same strain. Furthermore, the performance of recombinant HtrA2 in removing bloodstain from cotton fabric was tested.

## 2. Materials and Methods

### 2.1. Bacterial Strains, Plasmids and Culture Media

*Haloarcula* sp. TG1, an extreme haloarcheon, was isolated from Lake Tuz in Turkey in a previous study [20]. The plasmids pGEM-T^®^ Easy (Promega, Madison, WI, USA) was used for the cloning of *htrA2*, and pET28a(+) (Novagen, Madison, WI, USA) was used for the production of His-tagged HtrA2. *Haloarcula* sp. TG1 strain was grown in Sehgal–Gibbons (SG) [22] culture media at 37 °C with 200 rpm shaking. *E. coli* DH5α and BL21(DE3) strains were used for gene cloning and protein expression, respectively. *E. coli* cells were grown in Luria broth (LB) medium at 37 °C with 200 rpm shaking.

### 2.2. Molecular Cloning

After growing *Haloarcula* sp. TG1 in SG medium, DNA isolation was performed using the PureLink™ Genomic DNA Mini Kit (Invitrogen, Thermo Fisher Scientific, Waltham, MA, USA). To amplify the serine protease gene of the TG1 strain, forward primer (5′-ggatccatgttaacagccctctgtgatt-3′) and reverse primer (5′-aagcttcctcggatacgtctcggtta-3′) were designed using the *htrA2* (*HAH_5198*) gene sequence of *Haloarcula hispanica* ATCC 33960. The 1.1 kb gene was amplified by PCR using *Taq* DNA polymerase, the amplicon was ligated to the pGEM-T Easy vector, and the ligation product was transferred to *E. coli* DH5α. After the TG1 *htrA2* gene was confirmed by Sanger sequencing, it was cloned into the pET28a(+) vector and transferred to *E. coli* BL21(DE3) cells [23].

### 2.3. Phylogenetic Analysis and In Silico Protein Characterization

The nucleotide sequence of *htrA2* from the TG1 strain and the corresponding HtrA2 amino acid sequence were compared with those from different strains and species from eukaryotes and prokaryotes using the Basic Local Alignment Search Tool (BLAST) algorithm (https://blast.ncbi.nlm.nih.gov/Blast.cgi, accessed on 9 February 2026), and alignment was performed with ClustalW using MEGA v12 software [24]. Subsequently, a phylogenetic tree was constructed using the Neighbor Joining method. Motif analysis using the amino acid sequences of HtrA2 proteins was performed via the MEME Suite v5.5.9 tool (https://meme-suite.org/meme/tools/meme, accessed on 9 February 2026). The p*I* value and molecular weight of TG1 serine protease were calculated using the Expasy portal (https://web.expasy.org).

### 2.4. Production and Purification of HtrA2 Protein

When the OD_600_ value of the recombinant *E. coli* BL21 carrying pET-*htrA2* reached 0.5, gene expression was induced with 1 mM IPTG and incubated at 18 °C at 200 rpm for 16 h. Following incubation, the cultures were centrifuged at 4 °C and 6000× *g* for 15 min. Subsequently, the resulting pellets were incubated at −86 °C for 30 min and then thawed at room temperature. For protein purification, 5 mL of LEW buffer (50 mM NaH_2_PO_4_, 300 mM NaCl, pH 8.0) was added to the samples, followed by the addition of beta-mercaptoethanol (β-ME) to a final concentration of 28.6 mM. The mixture was then homogenized by pipetting/vortexing on ice and sonicated. The sonicated samples were centrifuged at 15,000 rpm for 15 min at 4 °C, and the resulting supernatants were passed through a Protino Ni-TED 2000 column (Macherey-Nagel, Dueren, Germany) to purify the recombinant HtrA2 protein. The purity of the samples was checked by sodium dodecyl sulfate (SDS)-polyacrylamide gel electrophoresis (PAGE) analysis [25]. An Amicon Ultra-15 30K centrifuge filter (Sigma-Aldrich, St. Louis, MO, USA) was used to concentrate the recombinant protein. Protein concentrations were determined by the Bradford method [26].

### 2.5. Zymogram Analysis

After purification, the catalytic activity of serine protease HtrA2 was analyzed qualitatively using zymogram. The 1% casein as a substrate was pre-mixed with SDS-polyacrylamide during gel preparation. Electrophoresis was performed after loading the serine protease onto the gel. The gel was then washed three times in renaturation buffer (2.5% Triton X-100, 1 M Tris-HCl, pH 7.5, 1 M CaCl_2_, 2 M ZnSO_4_) for 20 min. Following washing, the gel was incubated in reaction buffer (0.05% Triton X-100, 1 M Tris-HCl, pH 7.5, 1 M CaCl_2_, 2 M ZnSO_4_) at 50 °C for 24 h. Finally, the gel was stained with 0.15% Coomassie Brilliant Blue for 1 h at room temperature [27].

### 2.6. Analysis of Protease Activity

Protease activity determination was performed by optimizing the procedure of Pathak and Sardar [28]. An amount of 1 mL of enzyme (5 mg/mL) was mixed with 1 mL of substrate (1% casein in 50 mM phosphate buffer, pH 8.0) in a test tube and incubated at 50 °C for 30 min. After incubation, 3 mL of 10% tricarboxylic acid (TCA) solution was added to stop the reaction, mixed in a vortex, and left at room temperature for 15 min. Then, it was centrifuged at 13,000 rpm for 15 min, and the absorbance of the supernatant was measured at 280 nm. One unit (U) of protease activity was defined as the amount of enzyme required to release 1 μg of tyrosine in 1 min under the experimental conditions. The tyrosine standard curve was used to calculate the amount of tyrosine released as a result of enzymatic activity. The specific enzyme activity was expressed in U/mg [29].

The Michaelis–Menten constant (*K*m) and the maximum activity rate (V_max_) of HtrA2 protease from *Haloarcula* sp. TG1 were determined by a Lineweaver-Burk plot. Protease activity was measured under the standard assay conditions and by changing the casein concentrations (5 mM-50 mM).

### 2.7. Effects of Substrate, Temperature, pH, NaCl, Organic Solvent and Metal Ions on HtrA2 Activity

Casein, gelatin, and bovine serum albumin (BSA) were used as substrates at a 1% concentration. Subsequently, incubation temperatures of 30, 40, 50, 60, 70, 80, and 90 °C were tested in protease activity assays under standard conditions (50 °C, pH 8.0, 3.4 M NaCl) and in the presence of the specified substrates. The protease activity assay was performed at pH values of 5.0, 6.0, 7.0, 8.0, 9.0, 10.0, and 11.0 at the optimum temperature and 3.4 M NaCl concentration to define the optimum pH condition. Maximum protease activity was determined in the presence of 1.0, 2.0, 3.0, 4.0, and 5.0 M NaCl under the optimized temperature and pH conditions. To investigate the effect of organic solvents, inhibitors, and metal ions, 25% dimethyl sulfoxide (DMSO), 25% ethanol, 12.5% methanol, 10% acetone, 10% toluene, 10% benzene, 10 mM phenylmethanesulfonyl fluoride (PMSF), 10% SDS, and the metal ions Fe^2+^, Ca^2+^, Mn^2+^, Mg^2+^, and Zn^2+^ at a concentration of 5 mM were used [30]. First, the protease was pre-incubated in these solvents for 24 h, and activity measurements were performed under optimum experimental conditions (50 °C, pH 7.0, and 3.0 M NaCl). As a control, the untreated enzyme was used under the same conditions.

### 2.8. Thermal and pH Stabilities of HtrA2

For stability assays, protease samples were incubated for 24 h at temperatures of 30, 40, 50, and 60 °C and pH values ranging from 5.0 to 11.0 and were tested for activity studies. After incubation, residual enzyme activity was measured under the optimum conditions (50 °C, pH 7.0, and 3.0 M NaCl). The enzyme whose activity was measured directly under optimum conditions without any incubation step was considered to be 100%, and the activity values of others were calculated as relative percentages.

### 2.9. PHB Extraction

PHB extraction was performed according to the method reported by Taran [31]. After fermentation of the *Haloarcula* sp. TG1 in SG medium, 5 mL of culture was centrifuged at 10,000 rpm for 10 min. The resulting pellets were incubated with the same volume of sterile distilled water for 1 h at 37 °C. After centrifugation under the same conditions, the pellet was washed with 5 mL of acetone and ethanol, respectively, to extract the PHB. An amount of 5 mL of chloroform was added to the pellet, and a homogeneous mixture was obtained by pipetting. The mixture was kept in a water bath until the chloroform was completely evaporated. An amount of 5 mL of H_2_SO_4_ (95%) was added to the pellet, and the extraction was completed by incubating it in boiling water for 40 min.

### 2.10. Immobilization of Recombinant HtrA2 with PHB

PHB nanoparticles were used for immobilization. A solvent substitution technique using acetone and water was applied [32], in which pure recombinant HtrA2 solution was used instead of water. An amount of 0.5 g of powdered PHB was swollen by incubating in 50 mL of ethanol for 2 h at room temperature. Ethanol was removed and PHB was washed using 20 mL of 0.2 M sodium phosphate buffer (pH 7.0). PHB granules were added to the HtrA2 solution prepared at a concentration of 0.75 mg/mL in 0.2 M sodium phosphate buffer. The PHB granules with enzyme solution were incubated at 4 °C for 30 min at 150 rpm with stirring. The suspension was filtered twice through filter paper after adding 20 mL of 0.2 M sodium phosphate buffer (pH 7.0). It was then dried in a vacuum desiccator for 8 h. The activity and stability of the resulting immobilized and free enzymes were compared. Furthermore, to determine the reusability of immobilized HtrA2, which showed activity under optimum conditions, the same immobilized enzyme was removed from the medium and used consecutively in activity experiments 10 times [33]. The immobilized enzyme was recovered by centrifugation (8000× *g*, 20 min, 4 °C), washed once with 50 mM sodium phosphate buffer (pH 7.0), and reused in subsequent reaction cycles. The free enzyme was recovered using an Amicon Ultra-15 centrifugal filter unit (30 kDa MWCO, Sigma-Aldrich), centrifuged (8000× *g*, 20 min, 4 °C), and the retained fraction was reused under the same experimental conditions.

### 2.11. Removing Bloodstains from Cotton Fabric

The method described by Majithiya et al. [34] was used to evaluate the effectiveness of HtrA2 protease in removing blood stains from cotton fabric. In the experiments, 1 mg of protease was tested together with 1% commercial detergent (Ariel, Procter & Gamble, Cincinnati, OH, USA). Cotton fabrics were cut into 3 cm × 3 cm pieces; each piece was stained with 50 μL of blood and dried in an oven at 60 °C for 30 min. To fix the bloodstain, 100 μL of 2% formaldehyde was added to the fabrics and dried again at 60 °C for 30 min. Phosphate buffer alone (50 mM, pH 7.0), phosphate buffer containing pure HtrA2, phosphate buffer containing commercial detergent, and phosphate buffer containing HtrA2 and commercial detergent were added to the blood stains on four dried fabric samples, respectively. All samples were incubated for a total of 1 h at 50 °C, where the enzyme exhibits maximum activity, stirring every 10 min, and finally washed with distilled water. Stain removal efficiency was analyzed according to the color intensity remaining from the blood stain on the fabric using the ImageJ v1.54p (https://ij.imjoy.io) open source image processing program.

### 2.12. Statistical Analyses

All results are given as the mean and standard error of at least three independent experiments. To determine the statistical significance of the differences in activity and stability of HtrA2, Student’s *t*-test was used to compare means between two groups, while one-way analysis of variance (ANOVA) and Duncan’s multiple range test (DMRT) as a post hoc test was used to compare mean values of more than two groups. Statistical significance was defined as *p* < 0.05.

## 3. Results

### 3.1. Cloning, Sequencing and In Silico Analyses of the Serine Protease

For the cloning of the *Haloarcula* sp. TG1 serine protease gene, primers were designed according to the nucleotide sequence of the *H. hispanica htrA2* gene, amplification was performed by PCR, and the gene was cloned into the pGEMT-Easy vector. Subsequently, the 1122-base sequence obtained by nucleotide sequence analysis was submitted to GenBank and registered with the accession number PX933392. When this sequence was compared with other sequences in GenBank, it was found to be 99.38% similar to the *H. hispanica* strains N601 and ATCC 33960 *HISP_19165* and *HAH_5198* (*htrA2*) serine protease sequences and 99.29% similar to the SG26_17090 serine protease sequence of *Haloarcula* sp. CBA1115. The 373-amino acid sequence of HtrA2 from TG1 strain was compared to those in GenBank, and it was found to be 99.46% and 90.30% similar to the HtrA2 sequences of *H. hispanica* ATCC 33960 and *H. marismortui* ATCC 43049, respectively. The theoretical p*I* value of the TG1 HtrA2 protein was calculated as 4.84 and its molecular weight as 39.878 kilodaltons (kDa).

**Figure 1:**
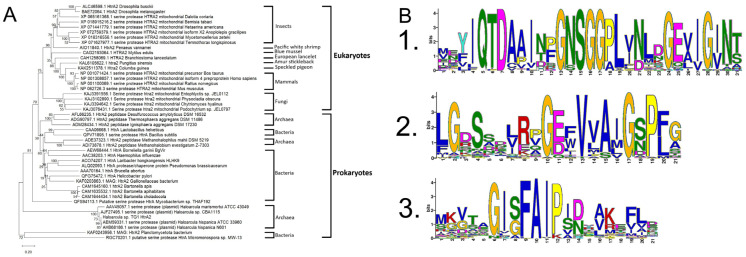
Phylogenetic tree (**A**) and conserved motifs (**B**) obtained using the amino acid sequences of HtrA2 proteins from diverse eukaryotic and prokaryotic organisms including that from *Haloarcula* sp. TG1.

The amino acid sequences of HtrA2 serine proteases from vertebrates, invertebrates, fungi, and bacteria were aligned, and a phylogenetic tree was constructed to examine their evolutionary relationships. It was noteworthy that these organisms were grouped according to the evolutionary relationships of their HtrA2 sequences. *Haloarcula* sp. TG1 HtrA2 enzyme was also found to be located on the same branch of the same phylogenetic tree as other *Haloarcula* spp. serine proteases (Figure 1A).

To discover the conserved patterns in aligned amino acid sequences, a motif analysis was performed. Three most common conserved motifs in the HtrA2 proteins were identified as (i) MEYIQTDAAINPGNSGGPLVNLDGEVIGINT, (ii) LGDSSDLRPGEFVVAIGSPFG and (iii) MKVTAGISFAIPIBRAKEFLD. The locations of these three motifs were observed to be very close to each other in HtrA2 sequences (Supplementary Figure S1). It was notable that these sequences contained highly conserved GNSGG motif and hydrophobic residues alanine (A), phenylalanine (F), valine (V), leucine (L), isoleucine (I) and glycine (G) in addition to neutral-polar residues asparagine (N), glutamine (Q), threonine (T) and serine (S), which is the nucleophilic amino acid in serine proteases (Figure 1B).

### 3.2. Purification of Recombinant HtrA2 Protein and Zymogram Analysis

**Figure 2:**
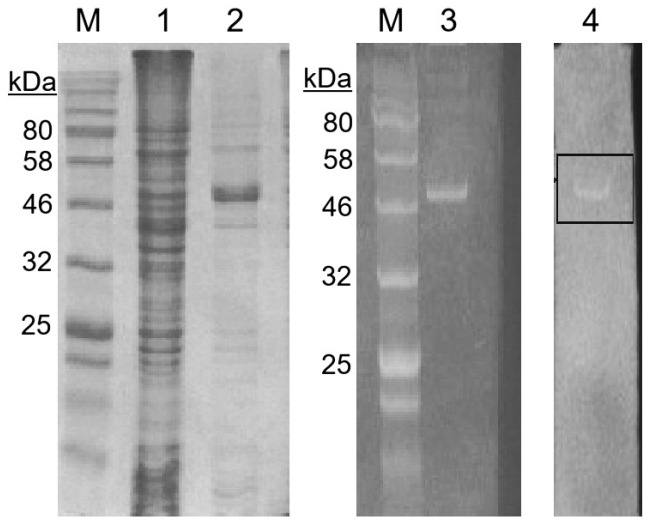
SDS-PAGE and zymogram analyses for the purification and enzymatic activity of recombinant HtrA2, respectively. Lanes 1 and 2 show the cell lysates of uninduced and IPTG-induced *E. coli* cultures, respectively. Lane 3 shows a single band of purified recombinant HtrA2 and lane 4 shows the enzymatic activity of HtrA2 on casein substrate as highlighted with a frame. M: Protein molecular weight ladder. Full photographs of the SDS-PAGE and zymogram analyses are presented in Supplementary Figure S2.

SDS-PAGE analysis showed that recombinant *E. coli* BL21 cells produced high amounts of HtrA2 serine protease after IPTG induction, and the recombinant enzyme was successfully purified (Figure 2). Recombinant HtrA2, along with His-tags, was observed to have a molecular weight of approximately 48 kDa on SDS-polyacrilamide gel. After purification with Ni-NTA columns and ultra centrifugal filters, 4.03 ± 0.28 mg/mL of recombinant HtrA2 enzyme was obtained from a 100 mL of IPTG-induced *E. coli* culture.

Purified HtrA2 was run on a SDS-polyacrylamide gel containing 1% casein as a substrate, renatured, and then enzymatic activity was monitored. As a result of the zymogram analysis, a substrate degradation zone was observed where the HtrA2 band was located (Figure 2).

### 3.3. Effect of Various Factors on the Protease Activity of HtrA2

#### 3.3.1. Substrate Effect

**Figure 3:**
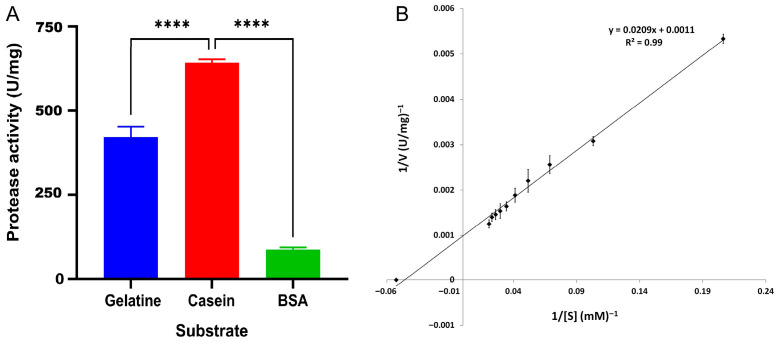
Enzymatic activity of recombinant HtrA2 on gelatin, casein and BSA as substrates (**A**) and Lineweaver-Burk plot used for the calculation of HtrA2 protease kinetics on casein substrate (**B**). ****: *p* < 0.0001.

To determine the effect of different substrates on protease enzyme activity and to identify the substrate yielding the best activity, casein, gelatin, and BSA were used at a concentration of 1%. According to the reactions carried out under standard conditions (50 °C, pH 8.0, 50 mM phosphate buffer and 3.4 M NaCl), the highest activity of recombinant HtrA2 was obtained with casein substrate with a value of 642 U/mg, followed by gelatin with 421 U/mg activity and BSA with 87 U/mg activity (Figure 3A). When HtrA2 protease kinetics on casein substrate were calculated using the Lineweaver-Burk plot (Figure 3B), the *K*m value was determined to be 19 mM casein, and the V_max_ value was 909 U/mg.

#### 3.3.2. Temperature Effect

**Figure 4:**
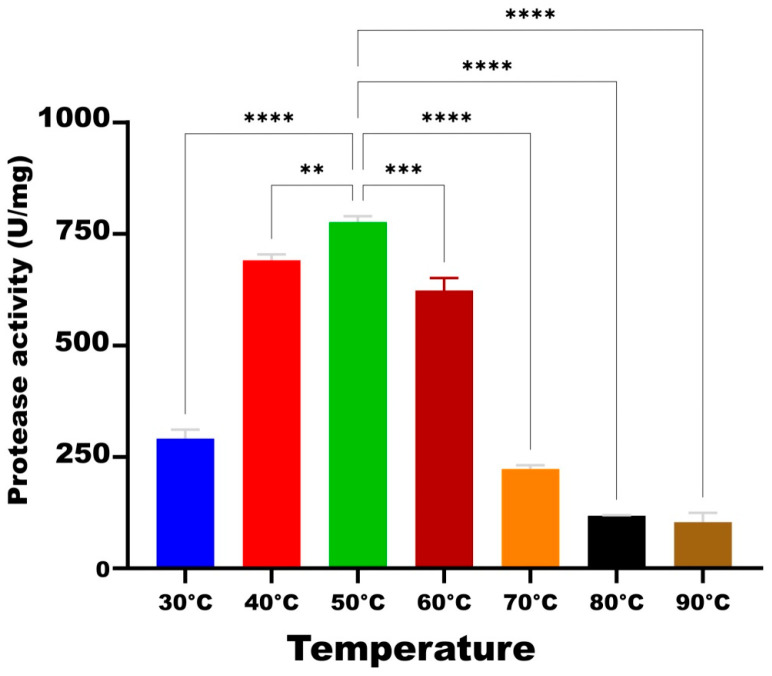
Effect of different temperatures on the enzymatic activity of recombinant HtrA2. **: *p* < 0.01, ***: *p* < 0.001, ****: *p* < 0.0001.

In the experiment evaluating the effect of temperature on the activity of HtrA2 serine protease under the standard conditions (pH 8.0, 50 mM phosphate buffer and 3.4 M NaCl), the highest activity (776 U/mg) was obtained at 50 °C. Compared to this temperature, 89% and 80.3% of the activity, respectively, was maintained at 40 °C (690 U/mg) and 60 °C (623 U/mg). On the other hand, HtrA2 activity decreased by 62.5% at 30 °C, 71.3% at 70 °C, 84.8% at 80 °C, and 86.7% at 90 °C compared to that at 50 °C (Figure 4).

#### 3.3.3. pH Effect

**Figure 5:**
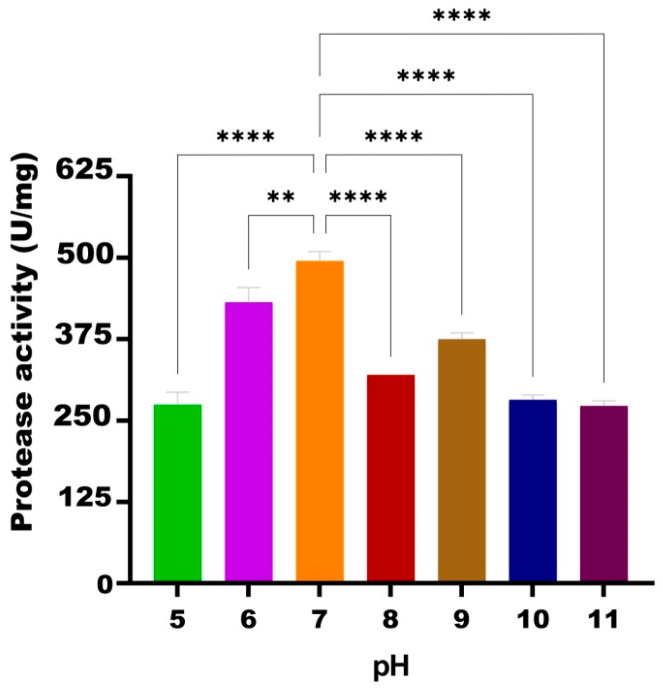
Effect of different pH values on the enzymatic activity of recombinant HtrA2. **: *p* < 0.01, ****: *p* < 0.0001.

In the experiment investigating the effect of different pH values on the TG1’s HtrA2 enzyme under standard conditions (50 °C, 50 mM phosphate buffer and 3.4 M NaCl), the highest activity was obtained at pH 7.0 (495 U/mg). This was followed by pH 6.0 (431 U/mg), pH 9.0 (375 U/mg), pH 8.0 (320 U/mg), pH 10.0 (281 U/mg), pH 5.0 (274 U/mg), and pH 11.0 (272 U/mg). Compared to the activity of HtrA2 at pH 7.0, 87.1% of the activity was obtained at pH 6.0, 75.7% at pH 9.0, and 55–65% activity was maintained at pH 5.0, pH 8.0, pH 10.0, and pH 11.0 (Figure 5).

#### 3.3.4. NaCl Effect

**Figure 6:**
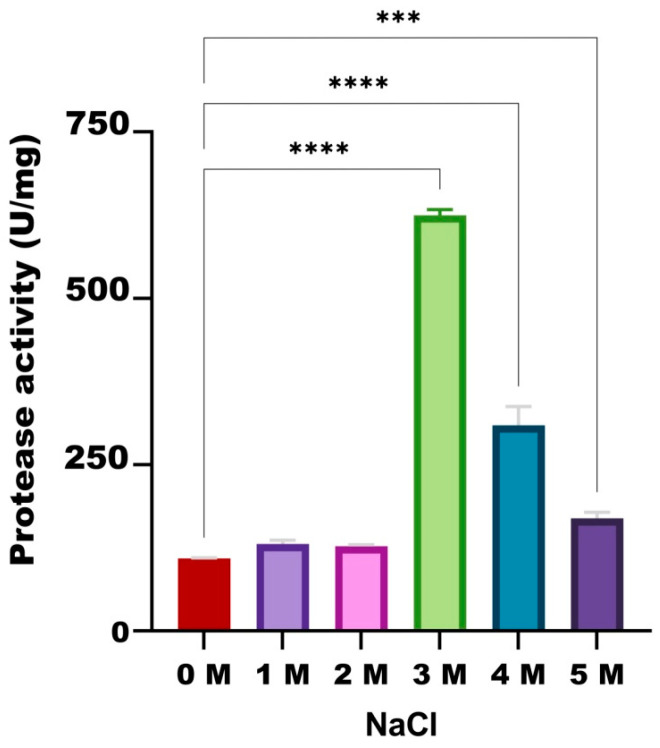
Effect of different NaCl concentrations on the enzymatic activity of recombinant HtrA2. The control group was the reaction with no NaCl (0 M). ***: *p* < 0.001, ****: *p* < 0.0001.

When the effect of NaCl salt on HtrA2 activity was examined under optimized conditions (50 °C, pH 7.0, 50 mM phosphate buffer), it was observed that the highest activity was obtained in the presence of 3.0 M NaCl, followed by 4.0 M NaCl (621 and 320 U/mg, respectively). While the protease activity was 110 U/mg in the absence of salt, enzyme activities slightly increased in the presence of 1.0 M, 2.0 M and 5.0 M NaCl (131, 128 and 174 U/mg, respectively) (Figure 6).

#### 3.3.5. Effect of Organic Solvents, Inhibitors, and Metal Ions

**Figure 7:**
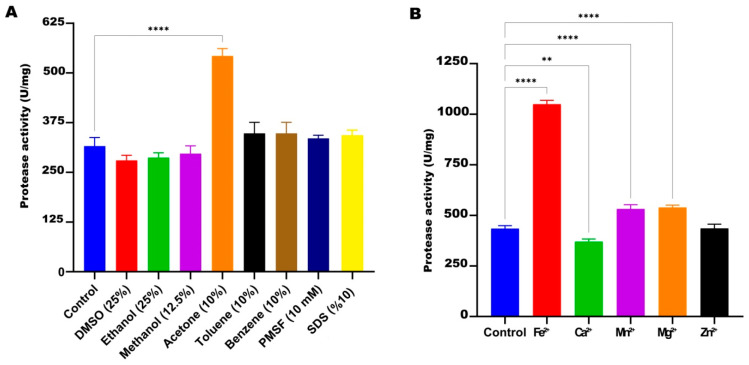
Effect of different organic solvents and inhibitors (**A**) and metal ions (**B**) values on the enzymatic activity of recombinant HtrA2. The control group was the reaction with no solvent, inhibitor or metal. **: *p* < 0.01, ****: *p* < 0.0001.

When the effect of various organic solvents and inhibitors on the protease activity of HtrA2 from TG1 was investigated under optimized conditions (50 °C, pH 7.0, 50 mM phosphate buffer and 3.0 M NaCl), it was observed that only acetone significantly increased activity (71%) compared to the control. DMSO, ethanol, methanol, benzene, toluene, PMSF, and SDS were found not to significantly affect protease activity compared to the control (Figure 7A).

In the experiment investigating the effect of various metal ions on recombinant HtrA2 protease activity, it was observed that Fe^2+^ ions increased the protease activity by 141.7% compared to the control. Mn^2+^ and Mg^2+^ ions also significantly increased the enzyme activity by 22.5% and 24.1%, respectively, compared to the control. On the other hand, it was observed that Zn^2+^ ions did not change HtrA2 activity, while Ca^2+^ ions caused a significant decrease in protease activity by 14.7% (Figure 7B).

#### 3.3.6. Temperature and pH Stability of HtrA2 Protease

**Figure 8:**
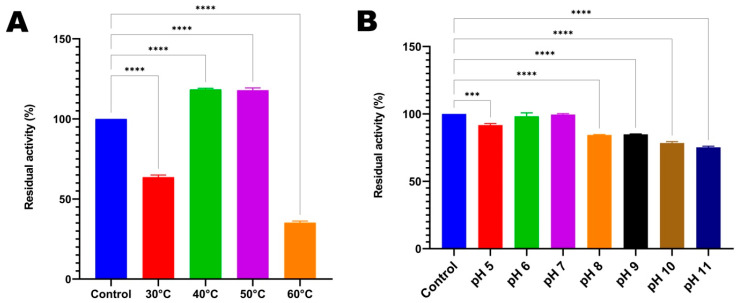
Stability of recombinant HtrA2 pre-incubated at different temperatures (**A**) and pH values (**B**) for 24 h. Residual activities are shown compared to the control group lacking the pre-incubation step. ***: *p* < 0.001, ****: *p* < 0.0001.

In the experiment conducted to determine the thermal stability of the HtrA2 serine protease enzyme of the TG1 strain, the enzyme was incubated for 24 h under different temperature conditions prior to activity measurement. Compared to the control, the enzyme activity increased by approximately 18% when HtrA2 was incubated at 40 °C and 50 °C. On the other hand, the protease activity decreased to 36.4% when the enzyme was incubated at 30 °C, and 64.8% loss was observed at 60 °C (Figure 8A).

In the experiment conducted to evaluate the pH stability of HtrA2, the enzyme was incubated for 24 h under different pH conditions prior to activity measurement. The best results were obtained when the enzyme was incubated at pH 6.0 and pH 7.0, with 98.1% and 99.5% of the preserved enzyme activity, respectively, compared to the control. On the other hand, although a statistically significant decrease occurred between pH 5.0 and pH 8.0–11.0 compared to the control, 91.7% of the enzyme activity was preserved at pH 5.0, and approximately 85–75% was preserved at pH 8.0–11.0 (Figure 8B).

### 3.4. Immobilization of HtrA2 Protease

**Figure 9:**
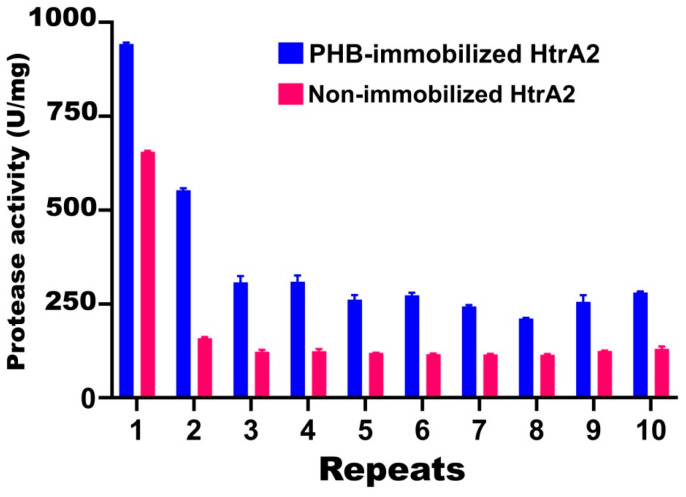
Immobilization of recombinant HtrA2 on PHB nanoparticles. Immobilized and non-immobilized (free) enzymes were used in 10 cycles to measure the enzyme activity under standard conditions.

It was determined that the activity of the recombinant HtrA2 protease of TG1 increased significantly when immobilized with PHB polymer, and the rate of activity loss in repeated use was less. In the first use, an activity of 944 U/mg was obtained with the immobilized enzyme, while an activity of 657 U/mg was obtained with the non-immobilized free enzyme. When the same enzymes were used a second time, the PHB-immobilized and non-immobilized enzymes showed an activity of 552 and 160 U/mg, corresponding to 41.5% and 75.6% loss of activity, respectively. It was determined that between the 3rd and 10th uses of the PHB-immobilized enzyme, a loss of activity of 67.1–77.5% occurred. In the same repeated uses of the non-immobilized enzyme, a loss of activity of 80.4–82.6% was observed (Figure 9).

### 3.5. Removal of Bloodstains from Cotton Fabric

**Figure 10:**
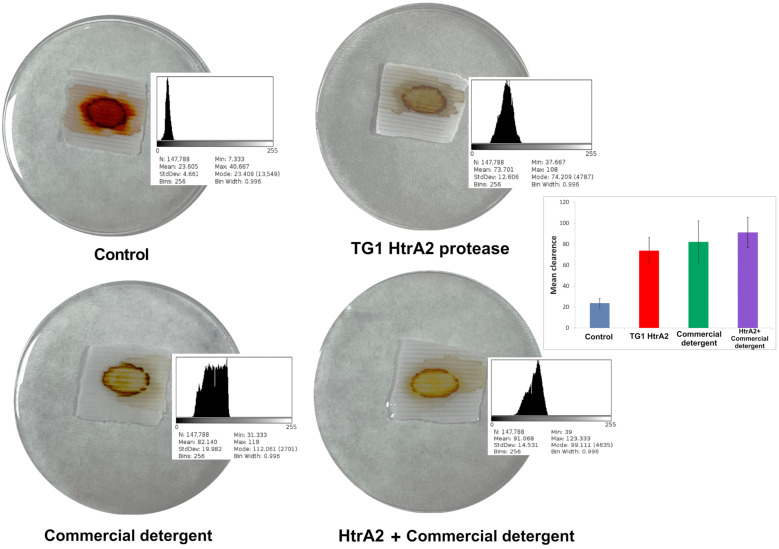
Removal of the bloodstain from cotton fabric by recombinant HtrA2 compared to a commercial laundry detergent (Ariel). The fabric in the control group was washed only with buffer solution. The areas containing the bloodstains were converted to histograms according to their color intensities, and the mean values are shown on the bar graphic.

To evaluate the effect of recombinant HtrA2 protease from *Haloarcula* sp. TG1 in a biotechnological application, its effectiveness in removing bloodstains from cotton fabrics was tested. After application of recombinant HtrA2, laundry detergent, and both together on the fabrics with bloodstains, their stain removal performances were evaluated with histograms. The average removal value was 23.6 in the control where only phosphate buffer was applied, while the average removal value was 73.7 in the application of HtrA2 protease, 82.1 in commercial laundry detergent, and 91.1 in the mixture of HtrA2 and commercial detergent. It was observed that HtrA2 protease and commercial detergent significantly removed the bloodstain from the fabric compared to the control, and the mixture of both showed slightly increased stain removal activity (Figure 10).

## 4. Discussion

Halophilic proteases, known for their stability and catalytic activity at harsh conditions, hold significant promise in industrial applications. They are primarily produced by haloarchaeal genera such as *Haloferax*, *Halobacterium*, *Halococcus*, *Natrinema*, and *Natrialba*, as well as certain halophilic bacteria including *Halobacillus*, *Bacillus* and *Pseudoalteromonas*. These proteases, used in the production of detergents, food, pharmaceuticals, textiles, and leather, also offer potential in environmental biotechnology [35,36,37].

HtrA2 serine protease is highly conserved and distributed among prokaryotes and eukaryotes. The mitochondrial origin of eukaryotic Omi/HtrA2 proteases strongly supports the idea that this protein belongs to a lineage of alpha-proteobacterial HtrA-like proteases [18,19]. Our phylogenetic analysis also showed that HtrA2 proteins are conserved between eukaryotes and prokaryotes. Our comprehensive characterization of HtrA2 from a halophilic archaeon will make a significant contribution to the literature on archaeal HtrA2 proteases.

In a previous study, we characterized the extracellular proteasome of the *Haloarcula* sp. TG1 [20]. Now, the gene encoding the HtrA2 serine protease enzyme of the TG1 strain was expressed in *E. coli*; recombinant HtrA2 was purified, characterized, immobilized, and its reusability was evaluated. It was reported that the TG1 extracellular proteasome showed activity at optimum pH 4.0, 50 °C, and 4.0 M NaCl, that DMSO and ethanol reduced enzyme activity by approximately 25%, and that other metal ions and inhibitors did not significantly affect activity. On the other hand, the purified recombinant HtrA2 protease was found to be active at optimum pH 7.0, 50 °C, and 3.0 M NaCl; Mn^2+^ and Mg^2+^ ions increased activity, but inhibitors had no significant effect. Acetone and Fe^2+^ ions also significantly increased HtrA2 activity, but their effects on the extracellular proteasome were not tested. The differing results obtained in the two studies are thought to be due to both the use of isolated HtrA2 and the different properties of the various proteases within the proteasome.

The number of studies on the molecular cloning, recombinant production and characterization of serine proteases from the halophilic microorganisms is limited. A serine endoprotease from the marine bacterium *Cobetia amphilecti* KMM 296 (CamSP) was heterologously produced using *E. coli* and purified. Recombinant CamSP showed 2369.4 and 1550.9 U/mg activities with the use of 1% BSA and casein as the substrates, respectively [38]. However, the activity of HtrA2 from TG1 was higher with casein substrate (642 U/mg) compared to that with BSA (87 U/mg). Another protease from *C. amphilecti* KMM 296 (CamClpP) was also produced recombinantly, and its activity was found to be 2824 U/mg using 1% casein [39]. The activities of proteases from the KMM 296 strain were much higher than that of HtrA2. On the other hand, HtrA2 showed significantly higher activity when compared to the recombinant protease obtained from the marine bacterium *Pseudoalteromonas phenolica* (Pph_Pro1), which had a maximum activity of 21.72 U/mg [37].

Proteolytic activity of CamSP was maximal at pH 5.6 and 7.4 while it was maintained between pH 4.0 and 10.0 [38]. Maximum CamClpP activity was obtained at pH 6.0–6.2 but its activity remained in a narrower range (pH 5.8 to 8.5) [39]. The highest activity for HtrA2 was at pH 7.0 (495 U/mg), and 87–55% of this activity was maintained at pH 6.0 to 11.0. The pH stability of HtrA2 was similar, with more than 90% activity maintained at pH 5.0–7.0, and approximately 85–75% was preserved at pH 8.0–11.0. The recombinant serine protease from *Marinobacter aquaeolei* MS2-1 was also active between pH 7.0 and 12.0, being the highest at pH 8.0 [40]. Similarly, recombinant serine proteases from *Oceanobacillus iheyensis* O.M.A_1_8 and *Haloalkaliphilic bacterium* O.M.E_1_2, isolated from salt enriched soil, was optimum at pH 8.0 and pH 10.0, respectively, while O.M.E_1_2 was highly active between pH 8.0 and 10.0 [41]. The optimum pH for Pph_Pro1 was pH 8.5–9.0 with a very low activity at pH 5.0 to 8.0 [37]. However, the activity of recombinant alkaline protease (APrBL) from *Bacillus lehensis* JO-26 from saline desert was maximal at pH 10.0, and it was maintained between pH 8.0 to 11.0 [42]. All of these halophilic proteases exhibited alkaliphilic properties, albeit to varying degrees, since they showed optimal activity and retained stabilities under alkaline pH conditions.

The optimum temperature requirement for HtrA2 was 50 °C. Although its activity remained high at 40 °C and 60 °C, its thermostability was low at 60 °C. APrBL also showed maximum activity at 50 °C and retained 73% of its activity at 80 °C [42]. The optimum temperatures for CamClpP [39] and CamSP [38] proteases were 45 °C and 50 °C, respectively. The optimum temperature for MS2-1 protease was also 50 °C, and its activity remained high between 40 and 70 °C [38]. For Pph_Pro1 protease, maximum activity was obtained at 90 °C and retained at 100% at 37 °C and 50 °C [37]. The activity of recombinant enzymes obtained from O.M.A_1_8 and O.M.E_1_2 was optimum at 37 °C; while 20–30% of the activity was maintained for 24 h at 37–50 °C, it completely disappeared at 60 °C [41]. As these reports show, most of the halophilic proteases are thermophilic.

HtrA2 showed a high NaCl requirement for its activity. Optimum enzyme activity was observed in the presence of 3.0 M NaCl, and half of this activity was retained at 4.0 M NaCl. O.M.A_1_8 and O.M.E_1_2 proteases preserved their 80–100% activity at 2.0–3.0 M salt, but the activity was lost totally after 3 h [41]. The activities of CamSP [38] and CamClpP [39] proteases were optimal at 0.3 M NaCl. The activity of APrBL was increased at the presence of 1.0–2.0 M NaCl [42]. Additionally, 2 mM NaCl did not decrease the activity of MS2-1 protease [40]. On the other hand, Pph_Pro1 protease showed its maximum activity in the absence of NaCl and the lowest activity at 3.0 M NaCl [37]. Although the sources of these proteases are halophilic microorganisms, NaCl requirements of the recombinant enzymes were reported to be a wide range.

Acetone, Fe^2+^, Mn^2+^ and Mg^2+^ ions increased HtrA2 activity. MS2-1 protease activity was also increased by Mg^2+^ but decreased by Fe^2+^. Additionally, Mn^2+^ and Mg^2+^ increased the activity of CamSP [38] but inhibited that of CamClpP [39], while acetone increased Pph_Pro1 activity [35]. However, Mn^2+^, Mg^2+^, and Fe^2+^ significantly inhibited the activity of APrBL [42]. Ca^2+^ ions caused a significant decrease in protease activity of HtrA2 while it increased the activity of APrBL [42], CamSP [38], CamClpP [39] and MS2-1 [40] proteases. On the other hand, Zn^2+^ ions, DMSO, ethanol, methanol, benzene, toluene, PMSF, and SDS did not significantly affect HtrA2 activity. However, ethanol completely deactivated Pph_Pro1 protease [37]. MS2-1 protease [39] and APrBL [42] were significantly inhibited by PMSF and Zn^2+^ but APrBL was stimulated by SDS, toluene and benzene [42]. Ethanol and Zn^2+^ increased CamSP activity [38] but inhibited CamClpP activity, in addition to SDS, while the latter was activated by PMSF [39]. Metal ions and inhibitors affect the activity of proteases in many varying ways, so that two proteases from the same strain can be influenced differently.

As a biotechnological application, we evaluated the usability of HtrA2 protease as a detergent additive. For this purpose, effect of HtrA2 protease was tested on the removal of a bloodstain from a cotton fabric. HtrA2 removed the bloodstain very efficiently, similar to the commercial laundry detergent. Moreover, we observed that using detergent and HtrA2 together gave a better result compared to using detergent and enzyme alone. Bloodstain removal activity of APrBL [42] and MS2-1 [40] proteases were also tested using the same procedure, and similar results were reported. Another application for the enzymes to use them in the industry is immobilization. In this study, PHB nanoparticles have been used for the immobilization of a protease for the first time, to our best knowledge. Furthermore, studies on the immobilization of purified halophilic proteases are quite limited. Khan et al. [43] recombinantly produced a subtilisin Carlsberg from *Bacillus haynesii*, isolated from a salt mine, and immobilized the purified enzyme on magnetic nanoparticles linked to glutaraldehyde and coated with chitosan. The thermostability of enzyme was increased 75% after immobilization, and 55% of immobilized enzyme activity and 50% of free enzyme activity was preserved after 10 cycles of reuse. In our study, 22.5% activity of the immobilized enzyme was retained after 10 cycles of reuse, which was 17.4 for the free enzyme. Also, protease activity of immobilized enzymes was higher in each cycle compared to free enzymes. Immobilization of HtrA2 improved the reusability of the enzymes in biotechnological applications.

## 5. Conclusions

Proteases are widely used in the industry, and halophilic proteases have a wide range of applications due to their resistance to harsh conditions. In this study, a serin protease, HtrA2, from *Haloarcula* sp. TG1, was heterologously produced in *E. coli*. Purified HtrA2 showed its optimum activity at 50 °C, pH 7.0 and in the presence of 3.0 M NaCl. Protease activity was maintained at a high level between 40 and 60 °C and pH 5.0 to 11.0. Moreover, acetone and Fe^2+^ significantly increased HtrA2 activity, and inhibitors such as PMSF and SDS did not inhibit enzyme activity. Purified HtrA2 was also immobilized on the PHB nanoparticles, which improved the reusability of the protease. Moreover, HtrA2 successfully removed the bloodstain from the cotton fabric. This comprehensive characterization of halophilic serine protease HtrA2 showed promising results regarding the usability of this enzyme in industrial applications.

## Data Availability

The original contributions presented in this study are included in the article/Supplementary Material. Further inquiries can be directed to the corresponding authors.

## Supplementary Materials

The following supporting information can be downloaded at: https://www.mdpi.com/article/10.3390/biom16030424/s1, Figure S1: The locations of conserved motifs in the HtrA2 sequences. Figure S2: Full photographs of the SDS-PAGE and zymogram analyses. The lane numbers correspond to those in Figure 2.

## Abbreviations

The following abbreviations are used in this manuscript:

BSA	Bovine serum albumin
DMSO	Dimethyl sulfoxide
HtrA	High-temperature requirement factor A
*K*m	Michaelis–Menten constant
PAGE	Polyacrylamide gel electrophoresis
PHB	Polyhydroxybutyrate
PMSF	Phenylmethanesulfonyl fluoride
SDS	Sodium dodecyl sulfate
U	Unit
V_max_	Maximum activity rate
