# Supplementary material for: Serine Protease HtrA2 from Halophilic Archeon Haloarcula sp. TG1: Heterologous Expression, Characterization and Immobilization
Source: Biomolecules. 2026 Mar 13;16(3):424. doi: 10.3390/biom16030424 (PMC13024417; doi:10.3390/biom16030424)
Supplement: Supplementary file 1 [file biomolecules-16-00424-s001.zip › Figure S1.pdf]

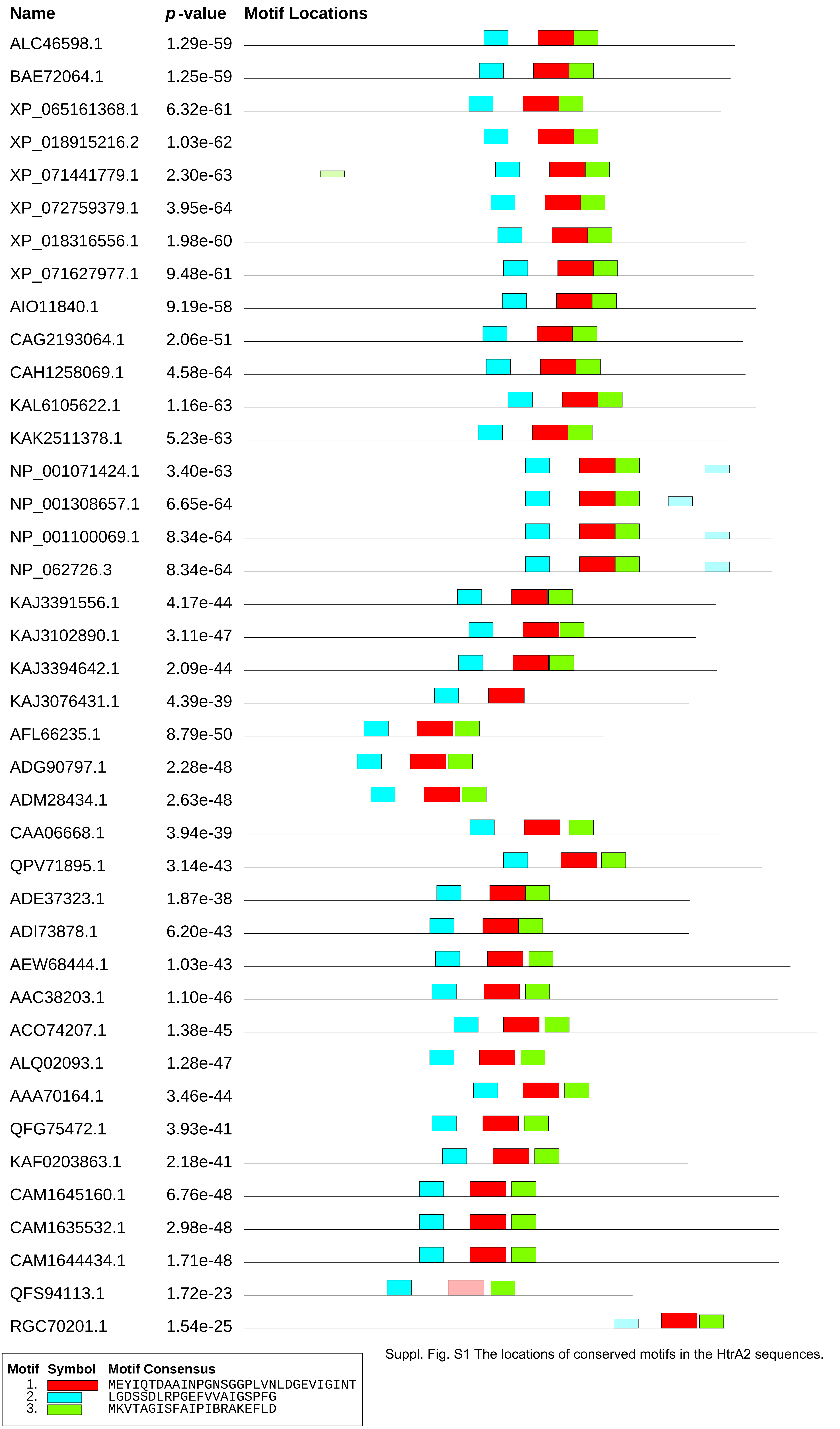

Suppl. Fig. S1 The locations of conserved motifs in the HtrA2 sequences.
