# Supplementary material for: Serine Protease HtrA2 from Halophilic Archeon Haloarcula sp. TG1: Heterologous Expression, Characterization and Immobilization
Source: Biomolecules. 2026 Mar 13;16(3):424. doi: 10.3390/biom16030424 (PMC13024417; doi:10.3390/biom16030424)
Supplement: Supplementary file 1 [file biomolecules-16-00424-s001.zip › Figure S2.pdf]

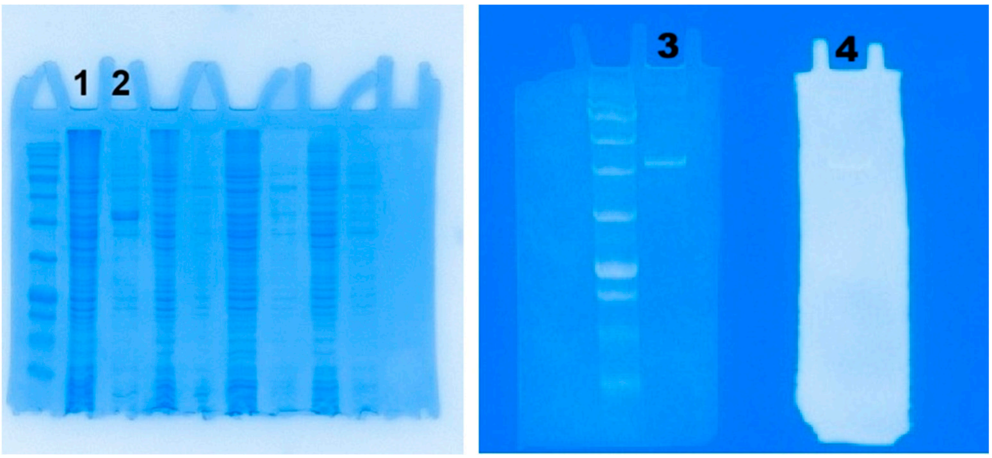

Figure S2. Full photographs of the SDS-PAGE and zymogram analyses. The lane numbers correspond to those in Figure 2.
